# 

**Published:** 1891-03-07

**Authors:** 


No. 232, Vol. IX.—March 7th, 1891.

[Registered at the General Post Office as a Newspaper for transmission in the United Kingdom and Abroad.]

Per Annum, 6s. 6d.; post free.

Monthly Parts, 6d.; post free 8d.

Ditto per Annum, 8s., post free.

### THE HOSPITAL

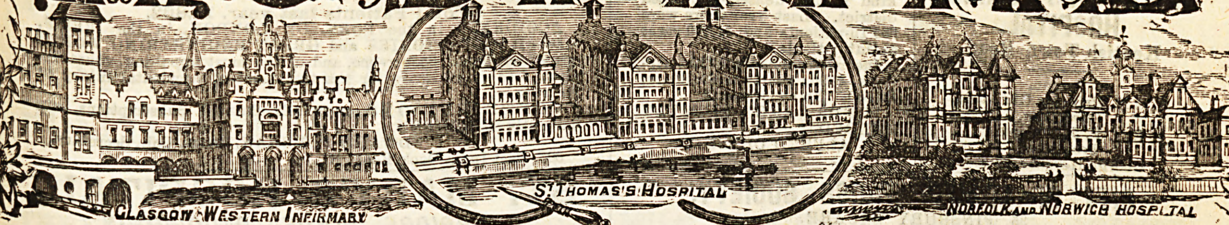

A WEEKLY JOURNAL OF

### Science, Medicine, Nursing and Philanthropy.

SATURDAY, March 7th, 1891.

**Body and Mind at Cambridge** - - - - - - - 331
**Passing Topics.**—On the Right Track - - - - - 332
**The Doctor's Armchair.**—Some Risks of Middle Age - 333
**The Microscope in Medicine.** By Frank J. Wethered, M.D.—VIII.—The Examination of Sputum - - - - 334
**Notes and News** - - - - - - - - - 336
**General Charities** - - - - - - - - - 337
**Scraps and Gleanings** - - - - - - - - 337
**Annotations.**—Mr. Nelson Hardy and Dr. Macnaughton Jones—A Suggested Snake-bite and Cholera Cure—Hospitals for All - - - - - - - - - 338
**The Practitioner's Mirror and Retrospect:—**
Medicine.—Aprosexia in Children—On Diseases of the Pancreas - - - - - - - - - 339
Surgery.—Fissures of the Tongue—Antiseptic Surgery - 340
Therapeutics.—Ignatia Amara - - - - - - - 341
New Drugs, Appliances, and Things Medical.—The Masticator—Horlick's Malted Milk - - - - - - 341
**The Lords' Committee** - - - - - - - 342
**Registration of Nurses** - - - - - - - 342
**The Student of Medicine.**—Leading Athletes—Editorial Notes—Appointments—Vacancies—Pass Lists—Athletics,

### EXTRA SUPPLEMENT—THE NURSING MIRROR.

**En Passant.**—Worcester City Institution—The Middlesex Hospital Nursing Institution—Miss Nightingale—A Ladies' Deputation—Presentation of Queen's Badges - cxxv
**Lectures on Surgical Ward Work and Nursing.** By Alex. Miles, M.B. Edin., C.M., F.R.C.S.E. Lecture XVI.—Dangers of Chloroform and Their Treatment - cxxvi
**Nursing Medals and Certificates.**—The London Hospital - - - - - - - - - - cxxvii
**For Reading to the Sick.**—Forsaken - - - - - cxxvii
**American News** - - - - - - - - - - cxxviii
**A Winter's Holiday.**—V.—A Spanish Town - - cxxviii
**Appointments** - - - - - - - - - - cxxix
**Everybody's Opinion.**—A Pension Fund for Servants—Personal Anecdotes—A Nurse's Hours - - - - - cxxix
**Princess Christian's Daughter** - - - - - - cxxix
**Off Duty.**—Nurse Hilary (*conclusion*) - - - - - cxxx
**There Was a Time** - - - - - - - - cxxx
**Amusements and Relaxation** - - - - - - cxxx

